# Supplementary material for: Dissociable encoding of evolving beliefs and momentary belief updates in distinct neural decision signals
Source: Nat Commun. 2025 Apr 25;16:3922. doi: 10.1038/s41467-025-58861-9 (PMC12032280; doi:10.1038/s41467-025-58861-9)
Supplement: Supplementary file 1 — Supplementary Information [file 41467_2025_58861_MOESM1_ESM.pdf]

Supplementary material for:

Parés-Pujolràs, E., Kelly, S. P., & Murphy, P. R. (2024). Dissociable encoding of evolving beliefs and momentary belief updates in distinct neural decision signals.

Authors: Elisabet Parés-Pujolràs [†1], Simon P. Kelly*[1], Peter R. Murphy*[2]

Affiliations:

[1]University College Dublin, Dublin, Ireland

[2]Maynooth University, Maynooth, Ireland
[†]Corresponding author

*These authors jointly supervised this work.

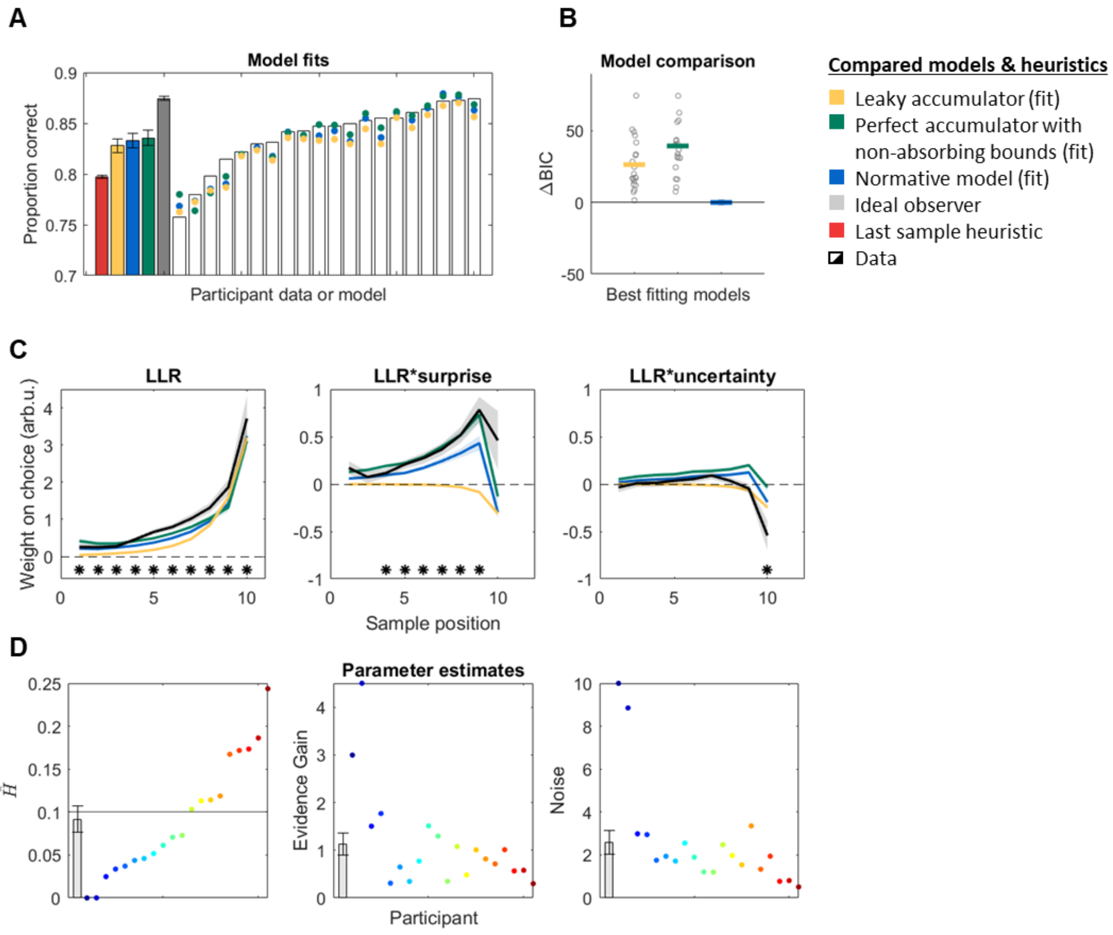

Supplementary Fig. S1. **The normative model provides the best fit to the data. A.** Proportion of observed and model-predicted correct responses. White bars and overlaid coloured points represent individual data and fits, respectively. Filled bars display grand-averaged data ± s.e.m.. **B.** Goodness of fit (BIC) of the three best fitting models, relative to the normative one. Overlaid dots indicate individual data. The normative model produced the best quantitative fit to the data (all $p < 0.05$, Bonferroni corrected, BIC paired t-tests). **C.** Observed and model-predicted (mean ± s.e.m.) weight on choice of each sample's LLR, and its interaction with change point probability (*surprise*) and uncertainty. *Asterisks along the bottom indicate samples where weighting significantly differed from zero ($p < 0.05$, two-tailed cluster-based permutation test). Several models can account for some of the normative model's behavioural implications. A leaky accumulator captured the increasing weight on choice of later samples but not how each sample's weight is modulated by surprise or belief uncertainty. A perfect accumulator with non-absorbing bounds, which effectively operates as a piecewise-linear approximation to the normative transfer function, was able to recapitulate all of the same key qualitative features of the data as the normative model. **D.** Estimated normative model parameters (hazard rate (*H*, *left*), evidence gain (*middle*) and noise (*right*)) for each individual participant (coloured *dots*), ordered as a function of H estimate, and at the group level(*bar graph;* mean ± s.e.m.). The average estimate of the hazard rate (H) did not significantly differ from the generative statistics (H = 0.1; $p = 0.59$, d = 0.12, 95%, CI = [0.05, 0.12] one-sample t-test).

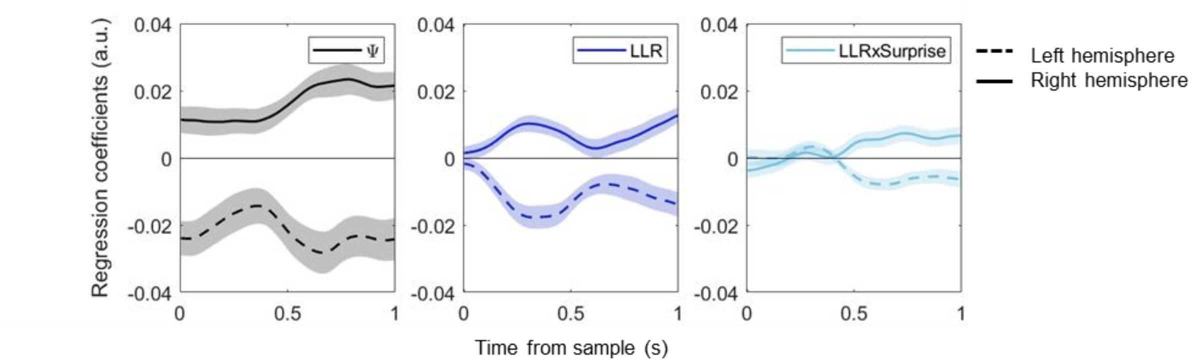

Supplementary Fig. S2. Standardized regression coefficients resulting from the analyses in *Eq. 9*, aligned to sample onset. Analyses were run on all electrodes separately, and regression coefficients were then averaged over the left- and right-cluster of electrodes over the motor cortex. Prior encoding and belief updates were implemented via modulation of power in both hemispheres. The prior (Ψ) and LLR take positive (negative) values when a right (left) hand response is favoured by the evidence, and the sign of the regression coefficients illustrates that these variables were associated with contralateral decreases and ipsilateral increases in motor beta power. Regressions were fitted separately for each sample, and then averaged over samples 2-10.

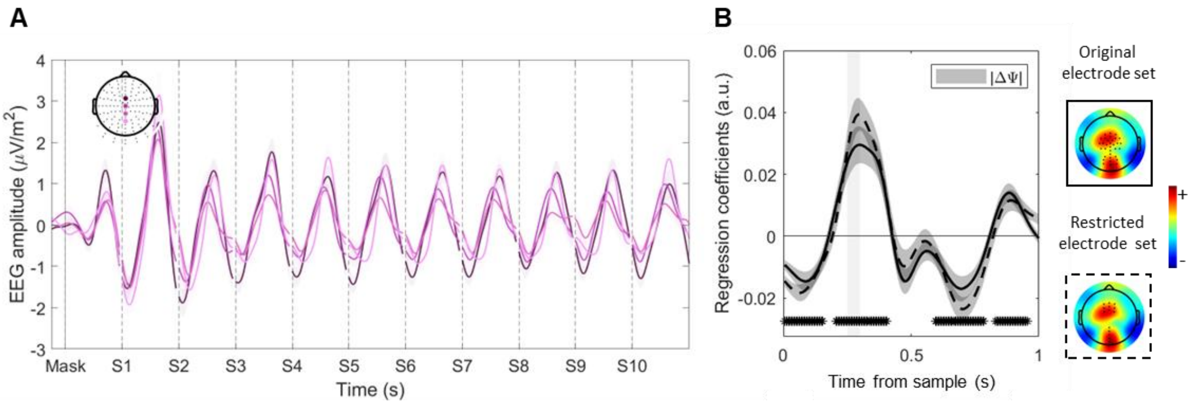

Supplementary Fig. S3. Reanalysis of data reported in Fig. 3 after applying 1Hz high-pass filter for residuals analysis. **A.** Grand-averaged ERP traces aligned to trial start in, in the highlighted electrodes (mean ± s.e.m.). Note the absence of positive drift in anterior channels (c.f. *Fig. 3A*). **B.** Standardised regression coefficients resulting from the analyses in *Eq. 12* averaged over samples 2-10 and aligned to sample onset (mean ± s.e.m.). Evoked responses in a pre-selected cluster of centroparietal electrodes tracked normative belief updates. *Asterisks indicate significant cluster periods (two-tailed cluster-based permutation test, $p < 0.05$). Topographies show whole-scalp regression coefficients [0.25-0.3s] post-sample, averaged over samples 2-10. Key effects of interest remain significant and consistent with the analysis reported in the main manuscript. The solid line shows results for the same selection of centroparietal electrodes used throughout the manuscript. To increase signal to noise ratio for the single-trial analysis, we extracted the residuals from a subset of electrodes where effective evidence encoding was maximal (*dashed line*).

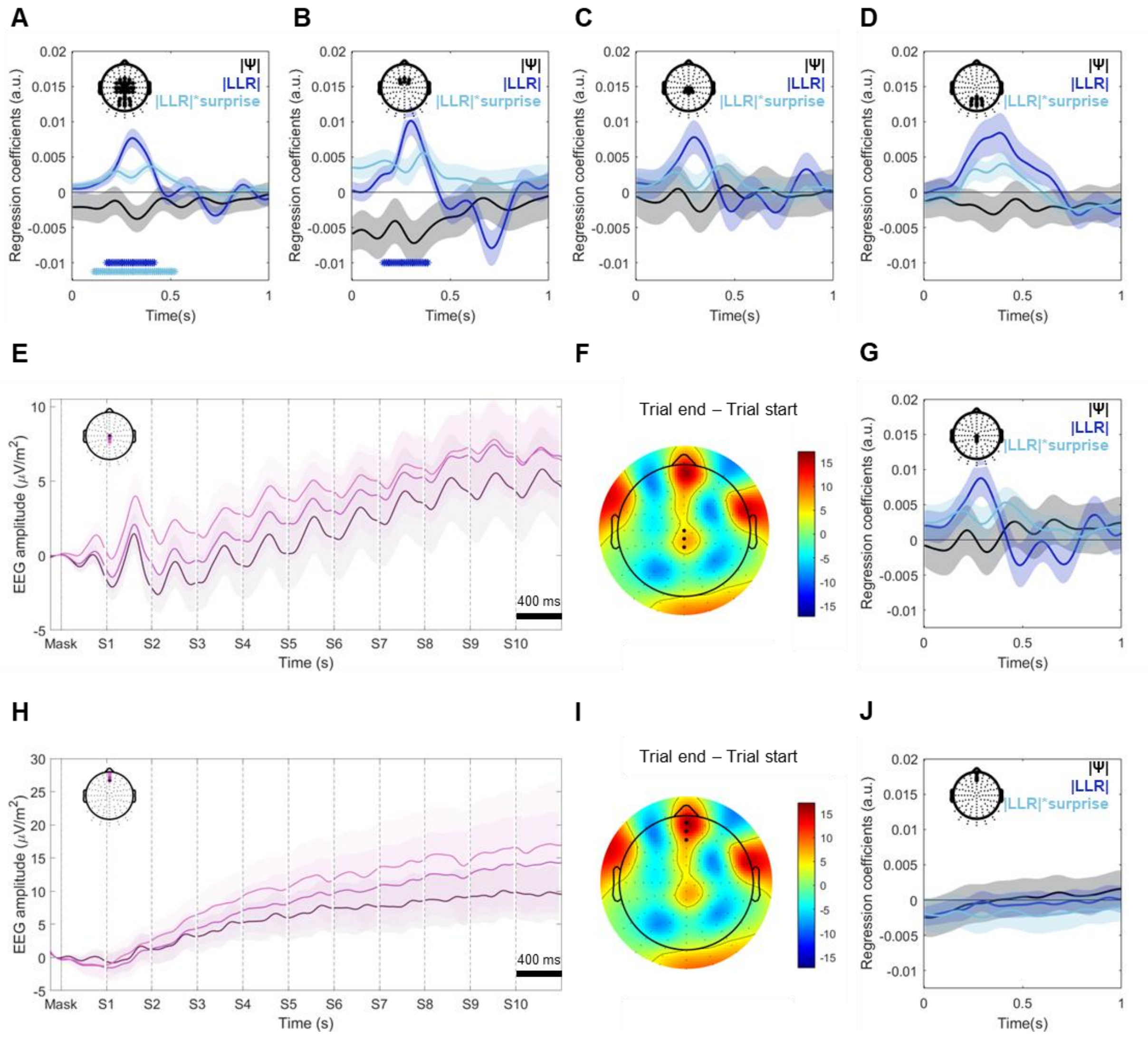

Supplementary Fig. S4**.** **A-D.** Regression coefficients for Eq. 11, reproducing the results of Fig 3B in various electrode subsets included in the original broad centroparietal channel selection. No significant prior effects emerged in any of those subsets, and all effects were qualitatively present in the various electrode subsets. **E,H.** Whole-trial ERPs, for central (E) and frontal (H) subsets of channels showing a strong positive drift from trial onset to trial end. We specifically picked those channels for analysis because this kind of positive-going activity is the one that most strongly resembles conventional continuous CPP traces, which have been shown to track the unsigned sum of accumulated evidence. **F,I.** Difference topographies between trial end (sample 10, [4-4.4s]) and trial start (Mask, [0-0.4s]). Positive values indicate channels with a positive-going drift, exhibiting higher amplitudes towards the end of the trial. **G,J.** Regression coefficients for the subset of central (G) and frontal (J) positive-going channels. Neither of these two subsets showed significant encoding of prior beliefs, indicating that the signal amplitude did not scale with the strength of unsigned prior beliefs. *In all panels, asterisks indicate significant cluster periods (two-tailed cluster-based permutation test, $p < 0.05$).

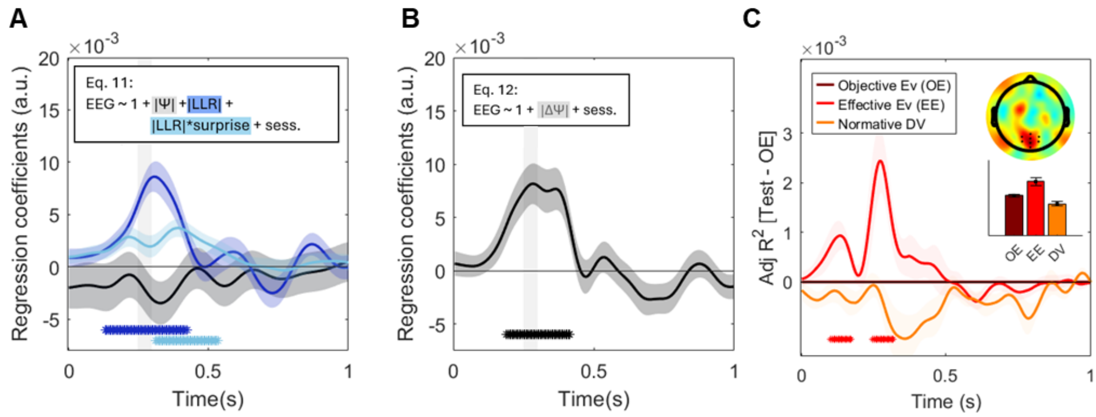

Supplementary Fig. S5. Replication of main analyses in Fig. 3 after excluding all samples where saccades or microsaccades were detected. Participants were instructed to maintain fixation at a central point throughout trials, and we used Independent Component Analysis (ICA) to remove both eye blink and horizontal eye movement artefacts from the signal. This should have removed (or at least heavily reduced) the potential for signal contamination, but it might not have removed saccade-related neural potentials (Yuval-Greenberg et al., 2008). To ensure that these potentials were not driving our results, we replicated our main analyses after excluding all samples with saccades or microsaccades in the 0.4s following sample onset. We used a conservative procedure for saccade detection based on velocity (30 deg/s), acceleration (8000 deg/s^2), and a minimum displacement of 0.5 degrees, following default Eyelink criteria and previous work (Engbert & Kliegl, 2003; Jackson et al., 2008). This ensured we also captured microsaccades. On average, participants broke fixation on at least one occasion on approximately 20% of the samples. We then repeated all analyses in Fig. 3B to D excluding all samples where either a saccade was detected or EyeLink data were poor quality. This resulted in the exclusion of an average of 30% of samples across participants from the regression analysis. We found that our key results **(A-B)** were unchanged by this exclusion, with CPP data still being best explained by an effective evidence (|ΔΨ|) model (**C**). These analyses suggest that our results were not driven by potentially overlapping saccade-related potentials. *In all panels, asterisks indicate significant cluster periods (two-tailed cluster-based permutation test, $p < 0.05$).

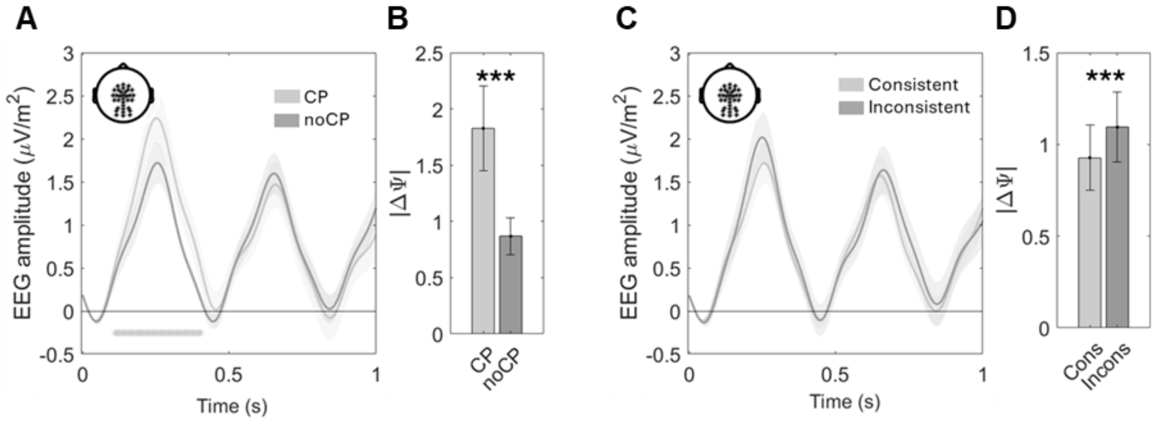

Supplementary Fig. S6. Sample sorting procedures that capture differences in |effective evidence| (|ΔΨ|) are accompanied by CPP modulations that scale with the magnitude of average |ΔΨ| differences. **A.** EEG amplitude at centroparietal channels following samples at a change point (CP) or in the absence of a change point (noCP). Change point samples evoked higher amplitude CPPs than no-CP ones (p = 0.018, two-tailed cluster-based permutation test). Data were baselined at sample onset to remove spurious drift-related baseline shifts. *Asterisks along the bottom indicate significant cluster periods for tests averaging over the highlighted electrodes. **B.** Mean (± s.e.m.) absolute effective evidence (|ΔΨ|) associated with change point (CP) were significantly higher ($p< 0.001$) than those associated with no-change point (noCP) samples. **C.** EEG amplitude at centroparietal channels following tokens consistent or inconsistent with prior beliefs (i.e. the LLR sign is either the same or different from the prior (Ψ) sign). Data were baselined at sample onset to remove spurious drift-related baseline shifts. **D.** Mean (± s.e.m.) absolute effective evidence (|ΔΨ|) associated with consistent or inconsistent samples.

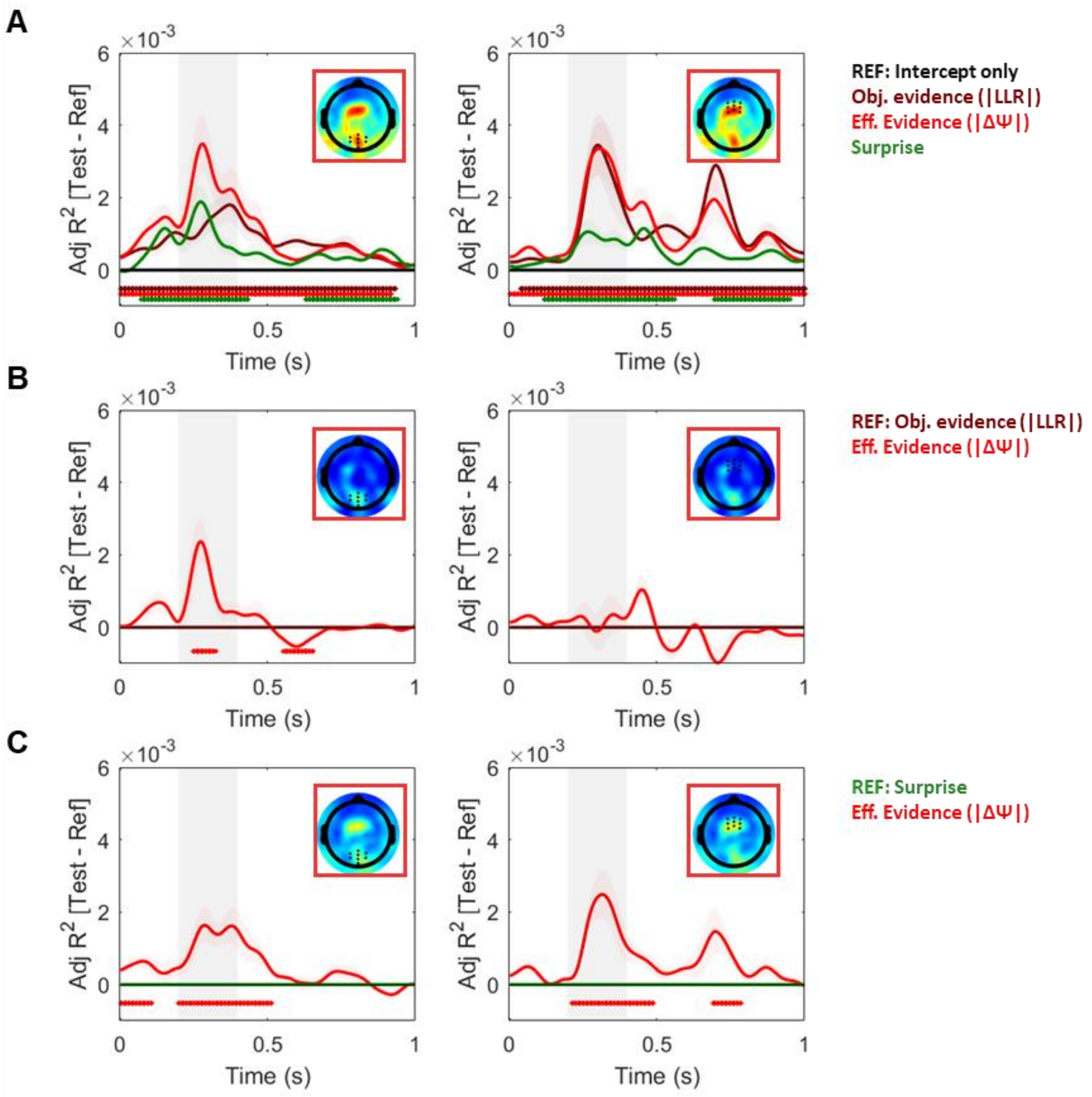

Supplementary Fig. S7. Comparison of single-regressor models at posterior (*left*) and anterior (*right*) electrode clusters, showing that the effective evidence model (|ΔΨ|) provides a better fit to EEG data than other models. Other work has suggested that CPP-like signals specifically encode forms of surprise in learning and predictive inference contexts (Mars et al., 2008; Nassar et al., 2019). However, we observed that the effective evidence model provided a better fit to the EEG evoked potentials than alternative models with two different forms of surprise as a the sole regressors: |LLR| (**B**), which corresponds to a low-level form of surprise on our task given the fact that high |LLR| stimuli are generally the least probable to occur; and change-point probability (referred to as 'surprise' above), which captures a high-level form of surprise conditional on the decision-maker's existing belief about the generative state (**C**). **A.** Adjusted R^2 square values of single-regressor models, compared to an intercept-only model, for posterior (*left*) and anterior (*right*) electrode selections where effects were maximal. The effective evidence model provided a better fit in both anterior & posterior electrodes. **B.** Adjusted R^2 values of the effective evidence model compared to the objective evidence one. The effective evidence model provided better fits in the posterior cluster of electrodes (*left*), and performed

equally well as the objective evidence model in the anterior cluster (*right*). **C.** Adjusted R^2 values of the effective evidence model compared to a surprise-only model. Topographies in each inset show the whole-scalp distribution of the difference between the effective evidence model and the reference model in each graph (**A,** intercept; **B,** objective evidence; **C,** surprise), over the timeframe indicated by the shaded area. Note: all models included a binary "session" regressor to control for any spurious differences between the two recording sessions.*Asterisks represent significant cluster periods (two-tailed cluster-based permutation test [0-1s], $p < 0.05$).

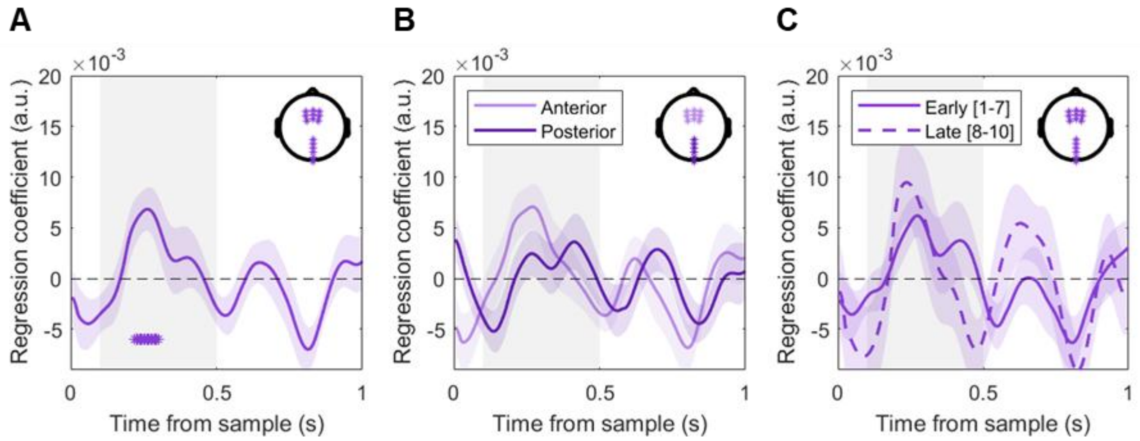

Supplementary Fig. S8. Normalised regression coefficients (mean ± s.e.m.) of *Eq. 17*, showing that variability in centroparietal responses influences motor beta lateralisation updates following each sample across all samples (***A***), in two electrode clusters (***B***), and sorted by sample times (***C***). *Asterisks indicate significant cluster periods within the times indicated by the shaded area (two-tailed cluster-based permutation test, $p < 0.05$).
