# Supplementary material for: Dissociable encoding of evolving beliefs and momentary belief updates in distinct neural decision signals
Source: Nat Commun. 2025 Apr 25;16:3922. doi: 10.1038/s41467-025-58861-9 (PMC12032280; doi:10.1038/s41467-025-58861-9)
Supplement: Supplementary file 2 — Reporting Summary [file 41467_2025_58861_MOESM2_ESM.pdf]

nature portfolio

Corresponding author(s): Elisabeth Parés-Pujolràs

Last updated by author(s): 04/03/2025

Reporting Summary

Nature Portfolio wishes to improve the reproducibility of the work that we publish. This form provides structure for consistency and transparency in reporting. For further information on Nature Portfolio policies, see our Editorial Policies and the Editorial Policy Checklist.

Statistics

For all statistical analyses, confirm that the following items are present in the figure legend, table legend, main text, or Methods section.

n/a	Confirmed	
☐	☒	The exact sample size (*n*) for each experimental group/condition, given as a discrete number and unit of measurement
☐	☒	A statement on whether measurements were taken from distinct samples or whether the same sample was measured repeatedly
☐	☒	The statistical test(s) used AND whether they are one- or two-sided<br>*Only common tests should be described solely by name; describe more complex techniques in the Methods section.*
☐	☒	A description of all covariates tested
☐	☒	A description of any assumptions or corrections, such as tests of normality and adjustment for multiple comparisons
☐	☒	A full description of the statistical parameters including central tendency (e.g. means) or other basic estimates (e.g. regression coefficient) AND variation (e.g. standard deviation) or associated estimates of uncertainty (e.g. confidence intervals)
☐	☒	For null hypothesis testing, the test statistic (e.g. *F*, *t*, *r*) with confidence intervals, effect sizes, degrees of freedom and *P* value noted<br>*Give P values as exact values whenever suitable.*
☒	☐	For Bayesian analysis, information on the choice of priors and Markov chain Monte Carlo settings
☒	☐	For hierarchical and complex designs, identification of the appropriate level for tests and full reporting of outcomes
☐	☒	Estimates of effect sizes (e.g. Cohen's *d*, Pearson's *r*), indicating how they were calculated

Our web collection on statistics for biologists contains articles on many of the points above.

Software and code

Policy information about availability of computer code

Data collection	EEG data were collected using a 128-channel BioSemi system. Behavioral data were collected with Matlab using stimulus presentation functions from Psychtoolbox 3.
Data analysis	All analysis code is available on the following GitHub repository: https://github.com/Elisabeth-Pares/ParesPujolras_Kelly_Murphy_2024.

For manuscripts utilizing custom algorithms or software that are central to the research but not yet described in published literature, software must be made available to editors and reviewers. We strongly encourage code deposition in a community repository (e.g. GitHub). See the Nature Portfolio guidelines for submitting code & software for further information.

Data

Policy information about availability of data

All manuscripts must include a data availability statement. This statement should provide the following information, where applicable:

- Accession codes, unique identifiers, or web links for publicly available datasets
- A description of any restrictions on data availability
- For clinical datasets or third party data, please ensure that the statement adheres to our policy

Behavioral and EEG data are now available on the following OSF repository: https://osf.io/nhqev/

Research involving human participants, their data, or biological material

Policy information about studies with human participants or human data. See also policy information about sex, gender (identity/presentation), and sexual orientation and race, ethnicity and racism.

Reporting on sex and gender	Five out of 20 participants self-identified as female and the remaining 15 self-identified as male when completing our consent form. No sex- or gender-based analyses have been performed on the data since the relevant hypotheses are not sex- or gender-specific.
Reporting on race, ethnicity, or other socially relevant groupings	No data on race, ethnicity or other socially relevant factors was collected.
Population characteristics	Twenty participants; age range 19-35 (M=23.1, SD=3.55).
Recruitment	19 of 20 participants were recruited through advertising around the campus of University College Dublin. Therefore it was a random selection, but contains mostly students in their 20s. The remaining participant was the last author of the manuscript. Participants were not assigned to condition-specific groups, and we believe that any self-selection bias should not impact the results.
Ethics oversight	All procedures were approved by the human research ethics committee of University College Dublin and participants were compensated for their time.

Note that full information on the approval of the study protocol must also be provided in the manuscript.

Field-specific reporting

Please select the one below that is the best fit for your research. If you are not sure, read the appropriate sections before making your selection.

☒ Life sciences ☐ Behavioural & social sciences ☐ Ecological, evolutionary & environmental sciences

For a reference copy of the document with all sections, see nature.com/documents/nr-reporting-summary-flat.pdf

Life sciences study design

All studies must disclose on these points even when the disclosure is negative.

Sample size	The sample size (N = 20) was determined a priori before beginning data collection. It is comparable or higher than previous studies using similar experimental designs (e.g. Murphy et al. 2021, Nat. Neuro; Wilming et al., 2020, Nat. Commun). Further, please note that all subjects attended 2 experimental sessions, and provided an average of 1440 trials per participant, which is notably higher than average in similar decision-making tasks.
Data exclusions	No subjects were excluded from any analysis. EEG data containing artefacts was either corrected using electrode- or single-trial level interpolation or excluded from analysis, as detailed in the manuscript methods. The artefact correction and trial rejection criteria were decided a priori and follow conventional practices in the field.
Replication	The computational modelling and motor beta lateralisation EEG results represent a replication of previous MEG work on which the current study is based (Murphy et al. 2021, Nat Neuro). However, the novel findings on the CPP have not yet been replicated beyond the current dataset. No attempts at replicating this experiment have been conducted beyond this dataset yet, but full details on the experimental procedure and analysis methods are provided to facilitate replication.
Randomization	Participants were all allocated into a single group for analysis, and thus randomisation of subjects was not necessary. Experimental stimuli were drawn at random from one of two generative distributions, and state changes were also determined at random.
Blinding	All subjects were analysed as part of a single group. Thus, blinding of experimenters was not necessary. Since participants performed one single task with no varying experimental conditions or difficulties, they did not need to be blinded to any experimental manipulations either.

Reporting for specific materials, systems and methods

We require information from authors about some types of materials, experimental systems and methods used in many studies. Here, indicate whether each material, system or method listed is relevant to your study. If you are not sure if a list item applies to your research, read the appropriate section before selecting a response.

Materials & experimental systems

n/a	Involved in the study
☒	☐ Antibodies
☒	☐ Eukaryotic cell lines
☒	☐ Palaeontology and archaeology
☒	☐ Animals and other organisms
☒	☐ Clinical data
☒	☐ Dual use research of concern
☒	☐ Plants

Methods

n/a	Involved in the study
☒	☐ ChIP-seq
☒	☐ Flow cytometry
☒	☐ MRI-based neuroimaging

Plants

Seed stocks

Report on the source of all seed stocks or other plant material used. If applicable, state the seed stock centre and catalogue number. If plant specimens were collected from the field, describe the collection location, date and sampling procedures.

Novel plant genotypes

Describe the methods by which all novel plant genotypes were produced. This includes those generated by transgenic approaches, gene editing, chemical/radiation-based mutagenesis and hybridization. For transgenic lines, describe the transformation method, the number of independent lines analyzed and the generation upon which experiments were performed. For gene-edited lines, describe the editor used, the endogenous sequence targeted for editing, the targeting guide RNA sequence (if applicable) and how the editor was applied.

Authentication

Describe any authentication procedures for each seed stock used or novel genotype generated. Describe any experiments used to assess the effect of a mutation and, where applicable, how potential secondary effects (e.g. second site T-DNA insertions, mosiacism, off-target gene editing) were examined.
